# Efficacy and safety of acupuncture combined with rehabilitation in the treatment of strephenopodia after stroke: A protocol for systematic review and meta-analysis

**DOI:** 10.1097/MD.0000000000028867

**Published:** 2022-02-18

**Authors:** Sisi Feng, Yihao Zhou, Mingzhi Tang, JuMei Wang, YuLan Lv, LiHua Gu

**Affiliations:** a Yunnan University of Traditional Chinese Medicine, Kunming, China; b Kunming Hospital of Traditional Chinese Medicine, the Third Affiliated Hospital of Yunnan University of Chinese Medicine, Kunming, China.

**Keywords:** acupuncture and rehabilitation, protocol, strephenopodia, stroke, systematic review

## Abstract

**Background:**

Strephenopodia is a common complication after stroke, which is easily neglected in the early stage of the disease and seriously affects the rehabilitation process of patients’ limbs, and brings huge security risks and family burden. A large number of studies have confirmed that acupuncture combined with rehabilitation (ACR) has a significant effect on strephenopodia after stroke (SAS), but there is still a lack of systematic scientific evidence to support this argument. In this systematic review, we aimed to evaluate the efficacy and safety of ACR in the treatment of SAS, to provide evidence-based medical evidence for the clinical treatment of the disease.

**Methods:**

We will search the following databases of 8 electronic databases from inception to January 2022: PubMed, EMBASE, the Cochrane Central Register of Controlled Trials (CENTRAL), Web of Science, China National Knowledge infrastructure (CNKI), Technology Periodical Database (VIP), WanFang Data, and China Biology Medicine (CBM). All relevant randomized controlled trials (RCTs) focus on ACR in the treatment of strephenopodia after stroke will be included. The primary outcome will be the Measurement of strephenopodia angle and Clinical Spasm Index Scale (CSI). The Secondary outcomes will include Holden Functional Walking Classification (FAC), Berg Balance Scale (BBS), and Modified Barthel Index Score (MBI). Two reviewers will independently conduct the Study selection and data extraction. The risk of bias will be evaluated according to the Cochrane tool. Risk ratio and 95% confidence intervals will be used to estimate the efficacy of treatment, and the grading of recommendations, assessment, development, and evaluation approach to rate the certainty of evidence. The data analysis will be analyzed using by RevMan5.4.

**Result:**

This study will provide a comprehensive evaluation of the efficacy and safety of ACR in the treatment of SAS, with a view of providing more reliable evidence-based solutions for SAS.

**Conclusions:**

The conclusion of this study will provide evidence to judge whether ACR is effective and safe in treating SAS.

**PROSPERO registration number:**

CRD42021290960.

## 1 Introduction

Stroke is an acute cerebrovascular disease with high morbidity, mortality and disability. According to the 2016 Global Burden of Disease Study, stroke caused by various cardiovascular and cerebrovascular diseases is the second leading cause of death worldwide. Cardiovascular and cerebrovascular diseases have caused about 17.6 million deaths worldwide, of which about 5.5 million died from cerebrovascular diseases.^[^1^,^^2^^]^ With the continuous progress of medical level, the mortality rate of stroke patients in the acute phase has decreased significantly, but its high disability rate has become a major problem vexing the medical community. According to studies, about 70%∼80% of stroke patients will leave varying degrees of limb dysfunction.^[3]^ Due to the irreversible damage of some high central nerves after stroke, the low central nerves lose effective regulation, cause hyperstretch reflex, present hemiplegia side muscle tone is abnormal, is the main reason that leads to limb motor dysfunction.^[^4^,^^5^^]^ At the same time, relevant studies have shown^[6]^ that the incidence of strephenopodia within 1 year after stroke is between 18% and 56%, which can lead to problems such as static and dynamic postural balance and walking. The main manifestations are increased tension of tibialis anterior muscle accompanied by varus, especially the muscle strength of peroneal short muscle is weakened, downward and inward twisting of affected side foot, varus angle greater than zero. When systemic tension or muscle tension increases, the strephenopodia is more obvious, and clinical studies have found that even if patients with strephenopodia can walk, they are prone to injury of ankle joint and surrounding soft tissue during walking, which seriously affects the quality of life and brings huge safety problems and family burden.^[^7^,^^8^^]^ As a natural state of the human body under normal conditions, balance is the foundation for maintaining normal walking and movement, whereas strephenopodia caused by muscle tension imbalance and abnormal muscle strength is the biggest obstacle to walking and balance function.

Presently, there is no magic drug for the rehabilitation of strephenopodia after stroke (SAS). Western medicine treatment is mainly based on local injection of Botulinum toxin A, which can improve varus symptoms to a certain extent, but its drug side effects are relatively large.^[9]^ Traditional Chinese medicine (TCM) believes that the occurrence of strephenopodia is mostly due to the obstruction of qi and blood circulation, the imbalance of yin and Yang, the contracture of the inner side of the limb and the relaxation of the outer side after stroke. In Eastern countries, acupuncture is widely used for post-stroke rehabilitation, and is one of the most important and effective treatment methods for SAS.^[^10^–^12^]^ On the contrary, with the continuous development of modern rehabilitation medicine, rehabilitation therapy has played an irreplaceable role in functional recovery after stroke.^[13]^ In recent years, more and more clinical studies have recognized that the relationship between acupuncture and rehabilitation therapy should promote each other. The combined application of acupuncture and rehabilitation can play a synergistic role to promote the relief of spasm, effectively improve the balance function, and strengthen the recovery of patients’ control, stability and coordination ability, so as to improve the curative effect of SAS.^[^14^,^^15^^]^ Although many clinical studies have reported the positive effects of ACR in the treatment of SAS, but scientific evidence is lacking. Therefore, this systematic review aims to evaluate the efficacy and safety of ACR in the treatment of SAS, and provide a better basis for clinical decision-making.

## 2 Methods and analysis

### 2.1 Study registration

This protocol of meta-analysis has been registered in PROSPERO with registration number as CRD42021290960. The results of this systematic review will be published according to the Preferred Reporting Item for Systematic Review and Meta-analysis (PRISMA)^[16]^ statement guidelines.

### 2.2 Inclusion criteria

#### 2.2.1 Type of studies

The included articles will be all randomized controlled trials (RCTs) focus on in the treatment of SAS. And there will be no restriction on language or publication status.

#### 2.2.2 Type of participants

All cases included in the trial will be patients with SAS, regardless of sex, age, educational background, nationality. However, participants must be mentally sound without cognitive impairment, mental disorder, and other severe underlying comorbidities.

#### 2.2.3 Types of interventions

Acupuncture (including needling, electroacupuncture, fire acupuncture, penetration acupuncture, blood-letting therapy, warm acupuncture, moxibustion, puncture cupping) combined with rehabilitation should be the major intervention in experimental group for the included articles. However, no specific criteria regarding acupoint selection, stimulus intensity, retention time, and treatment course is set.

#### 2.2.4 Types of comparisons

The group could gain guideline-recommended conventional treatment or acupuncture or rehabilitation or no treatment.

#### 2.2.5 Types of outcome measures

The primary outcome: Measurement of strephenopodia angle and Clinical Spasm Index Scale (CSI).

The Secondary outcomes: Holden Functional Walking Classification (FAC), Berg Balance Scale (BBS), and Modified Barthel Index Score (MBI).

### 2.3 Exclusion criteria

The exclusion criteria include:

1. The trails are not RCTs but theory studies, animal experiments, reviews or other unrelated literature researches.
2. The intervention of experimental groups is only acupunture or rehabilitation treatment without combined both of them.

### 2.4 Search strategy

Relevant studies published from the inception to January 2022 will be independently and systematically searched in appropriate databases by 2 researchers. The search shall be made in 4 English and 4 Chinese databases, include PubMed, EMBASE, the Cochrane Central Register of Controlled Trials (CENTRAL), Web of Science, China National Knowledge infrastructure (CNKI), Technology Periodical Database (VIP), WanFang Data, and China Biology Medicine (CBM). Relevant studies must have assessed the efficacy and safety of ACR on SAS. The search strategy that will be run in the PubMed and tailored to the other database when necessary is presented in Table 1. Besides, the references in related systematic reviews and clinical guidelines will also be searched to identify potential literature relevant to this study.

**Table 1:** The search strategy for PubMed.

No.	Search terms
#1	“strephenopodia”[Title/Abstract] OR “foot varus”[Title/Abstract] OR “pes supinatus”[Title/Abstract]
#2	“Stroke”[Mesh]
#3	“Strokes”[Title/Abstract] OR “Cerebrovascular Accident”[Title/Abstract] OR “Cerebrovascular Stroke”[Title/Abstract] OR “Apoplexy”[Title/Abstract] OR “Cerebral Stroke”[Title/Abstract] OR “Vascular Accidents, Brain”[Title/Abstract] OR “CAV”[Title/Abstract] OR “cerebral infarction”[Title/Abstract] OR “Ischemic Stroke”[Title/Abstract] OR “cerebral hemorrhage[Title/Abstract] OR “Hemorrhage Stroke”[Title/Abstract] OR“post-sroke” [Title/Abstract] #1 OR #2
#4	#2 OR #3
#5	“Acupuncture”[Mesh]
#6	“Acupuncture Treatment”[Title/Abstract] OR“Acupuncture and moxibustion”[Title/Abstract] OR“needling”[Title/Abstract] OR “electric- acupuncture”[Title/Abstract] OR “penetration acupuncture”[Title/Abstract] OR “fire needle”[Title/Abstract] OR “blood-letting therapy”[Title/Abstract] “Warm acupuncture”[Title/Abstract] OR “Puncture cupping”[Title/Abstract]
#7	#5 OR #6
#8	“Rehabilitation”[Mesh]
#9	“Habilitation”[Title/Abstract] OR “Routine rehabilitation treatment” [Title/Abstract] OR “rehabilitation training”[Title/Abstract] OR “modern rehabilitation”[Title/Abstract]
#11	#8 OR #9
#12	“Randomized Controlled Trial[Publication Type] OR “RCT randomized controlled”[Publication Type] OR “random allocation”[Title/Abstract] OR “allocation, random”[Title/Abstract] OR “randomized, controlled”[Title/Abstract] OR “clinical trial”[Title/Abstract]
#13	#1 AND #4 AND #7 AND #11AND #12

### 2.5 Study selection and data extraction

**Figure 1:**
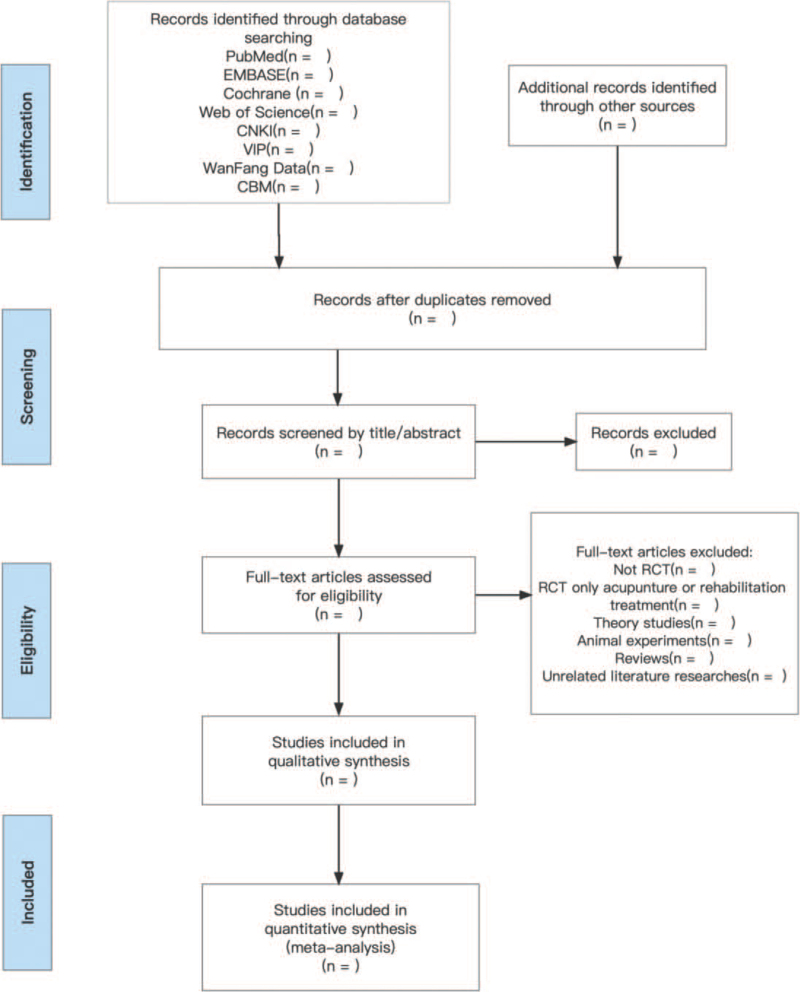
Flowchart of literature selection.

The data will be organized using the Endnote 20 (Endnote version 20.0, Thomas Reuters) referencing software literature management software. The process of study selection is shown in a flow diagram (Fig. 1). Studies meeting our inclusion criteria will be retrieved independently by 2 investigators who shall systematically screen through titles, abstracts, and keywords of the searched articles. The titles and abstracts for articles not meeting the eligibility criteria will be excluded. Relevant articles will be reviewed to extract significant findings. The extracted data will be organized and compiled by the 2 investigators, independently. Any disagreement regarding inclusion of some studies will be resolved by discussion and consensus between the 2 reviewers. If this failed, it shall be resolved by an arbitrator. Extracting data from the original article will be guided by a pre-determined data extraction form. The information include name of the first author, study location (country), year of publication, the total number of participants, study design, sample sizes, diagnostic criteria, the details on intervention, and control group, measurement for the main success indicators for positive outcome and cases of adverse events. If the data in a study are insufficient or ambiguous, the corresponding author will be contacted to provide additional information.

### 2.6 Assessment of risk of bias in included studies

The risk of bias will be assessed by 2 reviewers with the Cochrane “risk of bias assessment” tool and any differences will be resolved through consultation or the participation of a third reviewer. The risk of bias in included studies will be evaluated according to the following aspects and the assessments will be classified into 3 levels: low risk, high risk, and unclear risk:

1. random sequence generation;
2. allocation sequence;
3. blinding of participants and outcome assessors;
4. incomplete outcome data;
5. selective outcome reporting;
6. other bias.

### 2.7 Statistical analysis

Statistical analysis will be performed using the Review Manager 5.4 statistical software (Version 5.4. The Cochrane Collaboration, 2020). Risk ratio and 95% confidence intervals will be used to estimate the efficacy of treatment, and result for the meta-analysis will be presented using the forest plot. The *I* ^2^ statistic will be used to evaluate the statistical heterogeneity. When the *I* ^2^ >50%, the heterogeneity will be considered significant. Random-effects model will therefore be used for the pooled estimates and subgroup analyses. Conversely, if *I* ^2^ ≤50%, then the heterogeneity shall be considered not significant; thus the fixed-effects model shall be used in the subsequent meta-analysis. Two reviewers will independently use the grading of recommendations, assessment, development, and evaluation approach to rate the quality of evidence as high, moderate, low, or very low. When it comes to the situation that the data are insufficient for quantitative analysis, we will only perform a descriptive analysis.

#### 2.7.1 Subgroup analysis and sensitivity analysis

If significant heterogeneity will be detected, subgroup analysis will be carried out in accordance with the different treatments, control interventions and outcome measurements to explore potential sources of heterogeneity. If necessary, the sensitivity analysis will be used to assess the effect of each study on the random effects model. The sensitivity of the general combined effect of all outcome indicators is analyzed by the exclusion method. That is, all studies are excluded 1 by 1, and the remaining studies will be re-analyzed to determine the stability of the results. If there is no qualitative change in the combined effect showed in the results, the results are stable.

#### 2.7.2 Publication bias

The publication bias or heterogeneity will be evaluated using the symmetry of the funnel plot and Egger test. However, if there are <10 articles included, publication bias will not be explored.

#### 2.7.3 Grading the quality of evidence

Grading of Recommendations Assessment, Development and Evaluation (GRADE) method^[17]^ will be performed to evaluate the level of confidence in regards to outcomes. It is based on five key domains: risk of bias, consistency, directness, precision and publication bias. And the quality assessment results are divided into four levels: “high,” “medium,” “low,” and “very low.”

#### 2.7.4 Ethics and dissemination

This type of study is systematic reviews, and the entire study process does not involve the privacy information of individual patients, therefore does not require ethical approval.

## 3 Discussion

Strephenopodia as one of common complication after stroke patients, often neglected in the early disease, however, until the recovery of spastic gait, affected lower limb functional recovery was began to attach importance to treatment, seriously affecting the patient's gait, walking speed, walking ability and balance, causing the instability of walking of patients, increase the risk of falls, extend the body recovery time, thus affecting the quality of life of patients, the family and society to bring a heavy burden. At present, local injection of botulinum toxin A is the main treatment for strephenopodia in Western medicine, but long-term use is not recommended because of its toxic and side effects. Acupuncture combined with rehabilitation therapy, as a nondrug treatment mode, has become the most important comprehensive therapy for post-stroke rehabilitation and has been widely used in clinical practice.^[18]^ The combination of traditional acupuncture and modern rehabilitation technology can greatly shorten the clinical treatment period and improve the therapeutic effect. But as far as we know, there is still a lack of systematic scientific evidence to support this conclusion. This study will conduct a systematic review and meta-analysis of data from relevant RCTs to verify their effectiveness and safety, and provide evidence-based medical evidence for clinical treatment of the disease.

## Author contributions

All authors critically reviewed, revised, and approved the subsequent and final version of the protocol.

**Conceptualization:** Sisi Feng and LiHua Gu.

**Data collection:** Sisi Feng and Yihao Zhou.

**Data curation:** Sisi Feng, Yihao Zhou.

**Literature retrieval:** Mingzhi Tang and JuMei Wang.

**Resources:** Mingzhi Tang, JuMei Wang.

**Software operating:** Sisi Feng and Mingzhi Tang.

**Software:** Sisi Feng, Mingzhi Tang.

**Supervision:** YuLan Lv and LiHua Gu.

**Writing – original draft:** Sisi Feng and Yihao Zhou.

**Writing – review & editing:** LiHua Gu.
